# Synthesis of Glycolysis Inhibitor PFK15 and Its Synergistic Action with an Approved Multikinase Antiangiogenic Drug on Human Endothelial Cell Migration and Proliferation

**DOI:** 10.3390/ijms232214295

**Published:** 2022-11-18

**Authors:** Jana Zlacká, Miroslav Murár, Gabriela Addová, Roman Moravčík, Andrej Boháč, Michal Zeman

**Affiliations:** 1 Department of Animal Physiology and Ethology, Faculty of Natural Sciences, Comenius University in Bratislava, Ilkovičova 6, 842 15 Bratislava, Slovakia; 2 Department of Organic Chemistry, Faculty of Natural Sciences, Comenius University in Bratislava, Ilkovičova 6, 842 15 Bratislava, Slovakia; 3 Biomagi, Ltd., Mamateyova 26, 851 04 Bratislava, Slovakia

**Keywords:** PFK15 synthesis, sunitinib *L*-malate, HUVEC, angiogenesis, synergy

## Abstract

Activated endothelial, immune, and cancer cells prefer glycolysis to obtain energy for their proliferation and migration. Therefore, the blocking of glycolysis can be a promising strategy against cancer and autoimmune disease progression. Inactivation of the glycolytic enzyme PFKFB3 (6-phosphofructo-2-kinase/fructose-2,6-biphosphatase) suppresses glycolysis level and contributes to decreased proliferation and migration of cancer (tumorigenesis) and endothelial (angiogenesis) cells. Recently, several glycolysis inhibitors have been developed, among them (*E*)-1-(pyridin-4-yl)-3-(quinolin-2-yl)prop-2-en-1-one (PFK15) that is considered as one of the most promising. It is known that PFK15 decreases glucose uptake into the endothelial cells and efficiently blocks pathological angiogenesis. However, no study has described sufficiently PFK15 synthesis enabling its general availability. In this paper we provide all necessary details for PFK15 preparation and its advanced characterization. On the other hand, there are known tyrosine kinase inhibitors (e.g., sunitinib), that affect additional molecular targets and efficiently block angiogenesis. From a biological point of view, we have studied and proved the synergistic inhibitory effect by simultaneous administration of glycolysis inhibitor PFK15 and multikinase inhibitor sunitinib on the proliferation and migration of HUVEC. Our results suggest that suppressing the glycolytic activity of endothelial cells in combination with growth factor receptor blocking can be a promising antiangiogenic treatment.

## 1. Introduction

The alteration of metabolism and increased level of glucose metabolism in activated endothelial cells points to glycolysis as a possible target to inhibit pathological angiogenesis. Endothelial and tumor cells utilize glycolysis to metabolize glucose to lactate instead of oxidative phosphorylation, a phenomenon known as the Warburg effect, which provides an anabolic substrate to cover the demand for rapid cell proliferation and migration. The key regulator of glycolytic flux is the enzyme PFKFB3, which catalyzes the synthesis of F2,6P2 (fructose-2,6-bisphosphate), activating phosphofructokinase 1 (PFK-1), the rate-limiting key enzyme of glycolysis. In recent years, several glycolysis inhibitors have been developed, such as (*E*)-3-(pyridin-3-yl)-1-(pyridin-4-yl)prop-2-en-1-one (**3PO**) [1], (*E*)-1-(pyridin-4-yl)-3-(quinolin-2-yl)prop-2-en-1-one (**PFK15**) [2], and (*E*)-1-(pyridin-4-yl)-3-(7-(trifluoromethyl)quinolin-2-yl)prop-2-en-1-one (**PFK158**) [3]. Previously published data demonstrate that glycolysis inhibitors decrease glucose uptake and lactate production, simultaneously reduce the intracellular concentration of F2,6P2, and finally also reduce the level of glycolysis in the cells. Moreover, in our previous study, we confirmed that simultaneous administration of glycolysis inhibitor **3PO** and inhibition of the post-receptor signal cascade has a synergic inhibitory effect on the proliferation and migration of endothelial cells [4].

Generally, **PFK15** is considered to be a more effective glycolysis inhibitor than **3PO**, and its effectivity has been described in several studies [5,6]. In gastric cancer cell lines, **PFK15** induced cell cycle arrest by inhibiting the cyclin-CDKs/Rb/E2F-signaling pathway. Moreover, **PFK15** suppressed tumor growth by inducing the apoptosis of hepatocellular cancer cells and reducing the tumor vessel diameter [7]. Additionally, previously published data confirmed that chronotherapy represents an additional promising approach to glycolysis inhibition [5,8].

**PFK15** is not limited to cancer treatment but can also be related to understanding the activation of immune cells in chronic infection and autoimmune diseases. **PFK15** treatment reduces metabolic reprogramming in T-cells in vitro, leading to a delay in the onset of type I diabetes [9]. Therefore, the regulation of glycolysis metabolism in immune cells can be a new possible therapeutic target in autoimmune disease prevention. Additionally, uncontrolled angiogenesis contributes to the microvascular changes in diabetic patients, including the development of diabetic retinopathy [10]. Thus, angiogenesis inhibition is one of the potential targets in the diabetic nephropathy treatment [11].

The effectivity of **PFK15** was confirmed in several studies on different cell lines [2,12]; however, its synthesis has not yet been sufficiently described in the literature. The need for **PFK15** to be available on a larger scale, e.g., for in vivo assays, motivated us to develop the missing synthesis of this compound. Therefore, we here provide all necessary details for the synthesis of **PFK15** and its advanced characterization (e.g., by 2D NMR spectra), which are missing in the literature. To test the predicted antiangiogenic efficacy of **PFK15**, multiple assays were used. None of them were performed with a combination of **PFK15** and **sunitinib** before. The tests were performed on human umbilical vein endothelial cells (HUVEC), which provide a useful model system to study prospective antiangiogenic inhibitors or their synergy in combination with differently operating compounds.

## 2. Results

The antiangiogenic potential (endothelial cells growth, proliferation, and migration) of glycolysis inhibitor **PFK15** and multikinase inhibitor **sunitinib** was tested on HUVEC isolated from human umbilical cords.

### 2.1. Effect of PFK15 and Sunitinib on HUVEC Growth and Proliferation (IC_50_)

The IC_50_ concentration of HUVEC proliferation was determined as 2.6 µM for **PFK15** [8] and 1.6 µM for **sunitinib** (Table 1).

**Figure 1:**
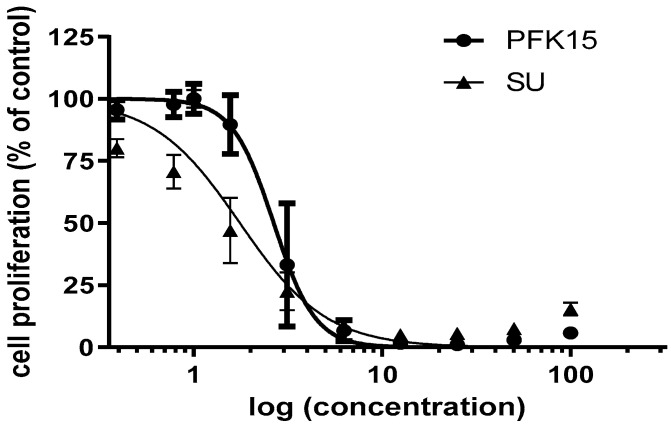
HUVEC response to **PFK15** ((*E*)-1-(pyridin-4-yl)-3-(quinolin-2-yl)prop-2-en-1-one) and **sunitinib** (SU) treatment after 72 h. Concentration values on the x-axis are on a logarithmic scale and are in µM.

As demonstrated in Figure 1, the HUVEC showed a different shape of response curve for **PFK15** and **sunitinib**, reflecting a higher sensitivity of HUVEC on **PFK15** concentration on the one hand, and a higher potential of **sunitinib** to block cell growth and proliferation at lower concentrations on the other, underlining its multikinase properties.

### 2.2. Effect of PFK15 and Sunitinib on HUVEC Migration (Wound Healing Assay)

**Figure 2:**
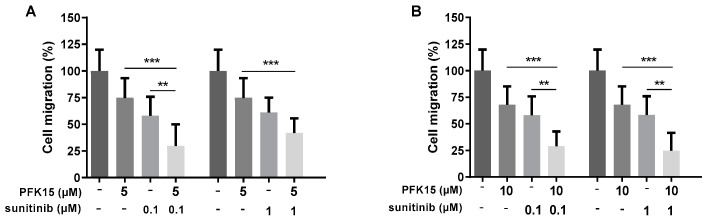
Evaluation of the administration of **PFK15** ((*E*)-1-(pyridin-4-yl)-3-(quinolin-2-yl)prop-2-en-1-one) and sunitinib, alone or combined, on HUVEC migration. HUVEC were treated with **PFK15** at 5 (**A**) and 10 µM (**B**) and sunitinib at 0.1 and 1 µM for 8 h and changes in cell migration were analyzed. Statistical differences among groups were determined by one-way ANOVA followed by Tukey’s post hoc test. Data are presented as the mean ± SEM of three independent experiments performed in quadruplicate (** *p* < 0.01; *** *p* < 0.001).

Cell migration was quantified after the cell treatment with **PFK15** and **sunitinib** alone or in combination. The combined action of **PFK15** (at 5 or 10 µM) with **sunitinib** (at 0.1 or 1 µM) more efficiently inhibited HUVEC migration compared to **PFK15** or **sunitinib** alone. Simultaneous administration of **PFK15** and **sunitinib** at specific concentrations clearly showed a synergistic effect on the inhibition of HUVEC migration (Figure 2; representative photos illustrating cell migration are available in Supplementary Material, Figure S8).

### 2.3. Effect of PFK15 and Sunitinib on HUVEC Proliferation

In the next experiments, we studied changes in HUVEC proliferation in the presence of **PFK15** and **sunitinib** in order to confirm their inhibitory and synergic effect. In the preliminary experiment, we applied **PFK15** at concentrations of 5 and 10 µM and sunitinib at 0.1 and 1 µM (similarly to the previous wound healing assay), alone or in combination, but in this case, the effects of inhibitors applied alone were too strong to see any combined effects (see the pictures in Supplementary Materials, Figure S9).

**Figure 3:**
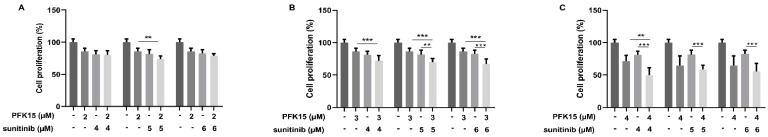
Effect of **PFK15** ((*E*)-1-(pyridin-4-yl)-3-(quinolin-2-yl)prop-2-en-1-one) and **sunitinib** administered alone or in combination on HUVEC proliferation. HUVEC were treated with **PFK15** at 2 (**A**), 3 (**B**), or 4 (**C**) µM and **sunitinib** at 4, 5, or 6 µM for 72 h. Changes in cell proliferation were recorded. Statistical differences among groups were determined by one-way ANOVA followed by Tukey’s post hoc test. Data are presented as the mean ± SEM of three independent experiments (** *p* < 0.01; *** *p* < 0.001).

Therefore, in the next experiments we selected lower concentrations of **PFK15** (2, 3, and 4 µM) and **sunitinib** (4, 5, and 6 µM) alone or in combinations. In these trials, both **PFK15** and **sunitinib** alone reduced cell proliferation compared to the control. Moreover, simultaneous administration of both inhibitors at specific concentrations showed a synergistic effect on cell proliferation compared to application of the inhibitors alone. **PFK15** at 3 and 4 µM in combination with **sunitinib** at 4, 5, and 6 µM most efficiently inhibited HUVEC proliferation (Figure 3B,C).

## 3. Discussion

The limited efficacy of the 1st generation of antiangiogenic therapy led to the development of selective PFKFB3 inhibitors that showed potential activity against the key glycolysis enzyme in different cell types, as well as in various animal models [2,5,6]. Recently, several glycolytic inhibitors were identified; among them, **PFK15** showed important antitumor efficiency. However, until now, no study has described the synthesis of this generally available glycolytic inhibitor in detail. Therefore, we performed an efficient synthesis of **PFK15** and confirmed its efficiency on activated HUVEC cells. Additionally, we observed more effective inhibition of HUVEC proliferation and migration using a combination of glycolysis inhibitor **PFK15** with multikinase inhibitor **sunitinib**, targeting different biological pathways and explaining the synergic effect of their inhibition.

**3PO** is one of the first synthesized blockers of glycolysis [1], and its effective synthesis has already been described by our research group [13]. Although its antiangiogenic potential was described in several studies, the concentration at which **3PO** is effective appears to be relatively high. Moreover, this high concentration is difficult to achieve in clinical studies because of the poor solubility of **3PO** in water [14]. Therefore, other inhibitors of PFKFB3 have been developed. Substitution of the pyridine-4-yl ring in the inhibitor **3PO** for the quinoline-2-yl group results in a more powerful inhibitor **PFK15**, which is also characterized by higher selectivity for the enzyme PFKFB3 [2]. The antitumor effect of **PFK15** was compared with the effect of chemotherapy drugs that are approved by the Food and Drug Administration (FDA) for the treatment of human cancer (e.g., irinotecan, temozolomide, and gemcitabine). The **PFK15** inhibitor suppressed pancreatic and colon adenocarcinoma growth, similarly to gemcitabine and irinotecan, while the effect of **PFK15** on glioblastoma growth was lower compared to the chemotherapeutic agent temozolomide [2]. The activity of the PFKFB3 enzyme can be regulated by several factors, including the AMPK signaling pathway, which can function as an upstream of the PFKFB3 regulator. It was confirmed that **PFK15** is able to inhibit AMPK and Akt-mTORC1 signaling in colorectal cancer cells [15], and thereby reduce tumor cell viability.

Limitations related to low inhibitor water solubility may be solved using nanocarriers or by chemical modification of the inhibitor structure [14]. Moreover, the low cytotoxic effect of glycolysis inhibitors on healthy cells [6], confirmed in several studies [16,17], suggests that the combination of a glycolysis inhibitor with other chemotherapy agents (e.g., multikinase inhibitor or cytostatic drug) may be an important strategy in advanced cancer treatment.

The additive inhibitory effect of the simultaneous administration of **3PO** and **sunitinib** on angiogenesis was demonstrated under in vivo conditions on a zebrafish embryo model, in which the formation of intersomitic vessels was evaluated [16]. Our previously published data [4] also confirmed that cells treated with the glycolysis inhibitor **3PO** at 10 µM and **sunitinib** at 1 µM, as well as 20 µM **3PO** in combination with **sunitinib** in a concentration range of 0.1–10 µM, significantly reduced cell proliferation compared to the inhibitors applied alone.

We determined the IC_50_ value for both **PFK15** and **sunitinib** (Figure 1) and have observed a significant decrease of HUVECs migration and proliferation after cell treatment with **PFK15** and **sunitinib** (Figure 2 and Figure 3). To confirm the synergic effect of the combined administration of both inhibitors, **PFK15** and **sunitinib** were applied at different concentrations. **PFK15** applied at 3 and 4 µM and **sunitinib** at 4, 5, and 6 µM more efficiently lowered cell proliferation compared to the inhibitors alone (Figure 3).

In addition to the simultaneous inhibition of the dominant metabolic pathway (e.g., by **PFK15**) and the activity on human kinase receptors (e.g., by **sunitinib**), there are also other possibilities to reduce cell metabolism and other physiological processes in cells. The inhibition of glycolysis in tumor cells may lead to the cells showing the increased preference for another metabolic pathway for ATP production, e.g., oxidative phosphorylation. The simultaneous inhibition of glycolysis and oxidative phosphorylation using metformin and 2-DG (2-deoxyglucose) showed an additive effect compared to the application of these inhibitors alone [18]. Another possibility is the use of phenformin, which was clinically tested in the treatment of diabetes, and oxamate, which acts as an inhibitor of lactate dehydrogenase enzyme activity. A synergistic effect was observed in the combined effect of phenformin and oxamate, where a significant antitumor effect was confirmed by simultaneous inhibition of complex I in mitochondria and LDH activity in the cytosol of the cells [17].

We used the IC_50_ value, which expresses the concentration of tested substance causing inhibition of cell growth by 50%, to determine the toxicity of **sunitinib** and the glycolysis blocker **PFK15**. The measured values show that HUVEC are more sensitive to **sunitinib** compared to **PFK15**. Similar results were obtained with bovine aortic endothelial cells (BAEC) at the IC_50_ concentration of 2.2 µM of **sunitinib** and 1.6 µM for HUVEC, respectively (Table 1). This reflects the multi-targeted tyrosine kinase inhibitory action of **sunitinib** compared with **PFK15**. The multikinase inhibitor **sunitinib** is used in the clinical treatment of several types of cancer and its effect has been documented by many published data [19,20]. The mechanism of action of **sunitinib** includes several signaling pathways, possessing receptor as well as non-receptor tyrosine kinases (e.g., VEGF-R, PDGF-R, CSFR, c-KIT, etc.) [21].

Consistent with our results, the different effectivity of **3PO** and **PFK15** was shown in a model of an immortalized human T-lymphocyte line (Jurkat cells) and non-small cell lung carcinoma cells (H522) after 48 h of treatment, with **PFK15** having a more pronounced effect than **3PO** [2]. Previously published data suggest that the effect of these inhibitors varies significantly depending on the cell type and origin (human vs. animal cells), degree of differentiation, inhibitor concentrations, and time of incubation [1,2,12,22]. Our previous study showed that **PFK15** reduced HUVEC and colorectal adenocarcinoma DLD1 cell proliferation at the same level, indicating that the same molecular targets are affected by this treatment [8]. However, compared to an animal model of BAEC, the effectivity of **PFK15** was quite different, as the IC_50_ value for BAEC was determined to be 7.4 µM. **3PO** showed similar results to **PFK15**, with an IC_50_ value of 6.9 µM, indicating that the effectivity of glycolysis inhibition depends on several additional factors.

The simultaneous administration of **PFK15** and the chemotherapeutic drug paclitaxel (PTX) showed a synergistic effect and may be an effective strategy in cancer treatment. Both drugs were carried to the cells by solid lipid nanoparticles encapsulated in membranes formulated by fusing breast cancer cell and activated fibroblast membranes [23]. The cytotoxic effect of PTX/**PFK15** in solid lipid nanoparticles was confirmed in vitro, as well as in vivo. In tumor-bearing mice, the monotherapy of nanoparticles with PTX or **PFK15** did not induce a significant therapeutic effect; however, mice treated with PTX/**PFK15** nanoparticles showed enhanced tumor inhibition. These data suggest that the dual targeting of cancer cell and cancer-associated fibroblasts (CAF) is more effective, as the energy supply from CAF to cancer cells is blocked by glycolysis inhibition [23].

In summary, we developed the missing synthesis of **PFK15** with the aim of making this glycolysis inhibitor broadly available in large amounts for in vitro and in vivo biological studies. Additionally, **PFK15** was also thoroughly physicochemically characterized. From a biological point of view, we proved sufficient sensitivity of HUVEC proliferation and migration to both inhibitors alone. More importantly, we have proven the synergistic effect on HUVEC proliferation and migration with the combination of **PFK15** and **sunitinib** at their appropriate concentrations. In future research, the pharmacokinetic properties of **PFK15** should be improved by developing more biologically active form of inhibitor, which can be tested either alone and/or in combination with other clinically used drugs in different pathophysiological conditions.

## 4. Materials and Methods

### 4.1. Chemistry

#### 4.1.1. General Conditions

The starting chemicals for syntheses were purchased from Sigma-Aldrich (St. Louis, MO, USA). Other chemicals and solvents were purchased from local commercial sources and were of analytical grade quality. The melting point was measured by a digital melting point apparatus Barnstead Electrothermal IA9200 and is uncorrected. ^1^H- and ^13^C-NMR spectra were recorded on a Varian Gemini (600 and 150 MHz, respectively); chemical shifts are given in parts per million (ppm); tetramethylsilane was used as an internal standard; and DMSO-*d*_6_ was used as the solvent. Infrared (IR) spectra were acquired by Fourier transform infrared spectroscopy (FT-IR) attenuated total reflectance REACT IR 1000 (ASI Applied Systems) with a diamond probe and MTS detector. Mass spectra were performed on a liquid chromatography mass spectrometer LC-MS using an Agilent Technologies 1200 Series equipped with mass spectrometer Agilent Technologies 6100 Quadrupole LC-MS. The course of the reactions was followed by thin-layer chromatography (TLC) (Merck Silica gel 60 F254), and a UV lamp (254 nm), and iodine vapors were used for visualization of the TLC spots.

#### 4.1.2. Synthetic Pathway to (*E*)-1-(Pyridin-4-yl)-3-(quinolin-2-yl)prop-2-en-1-one (**PFK15**)

**Scheme 1:**
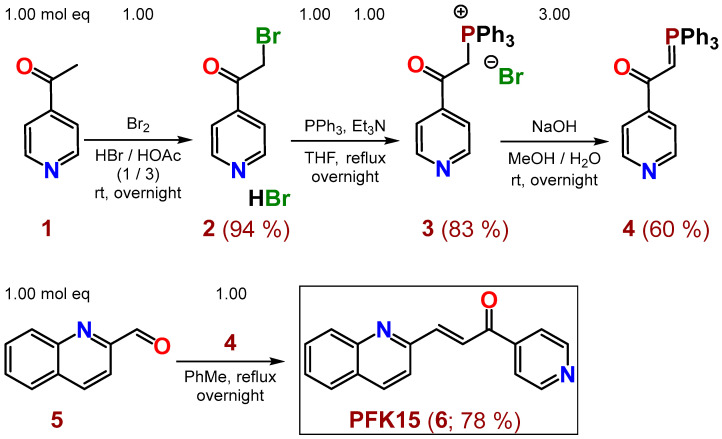
Synthesis of inhibitor **PFK15** (**6**), starting from commercially available 1-(pyridin-4-yl)ethan-1-one (**1**) and 2-quinolinecarboxaldehyde (**5**).

Inhibitor **PFK15** (**6**) was prepared in four reaction steps, according to Scheme 1. After bromination of commercially available 4-acetylpyridine (**1**) with bromine in a mixture of conc. acids HBr/HOAc (1/3, respectively) at room temperature (rt), the hydrobromide of 2-bromo-1-(pyridin-4-yl)ethan-1-one (**2**) was obtained in 94% yield. Salt **2** was converted to compound **3** in 83% yield by PPh_3_ and Et_3_N in THF under reflux overnight. Compound **4** was prepared from salt **3** in 60% yield by NaOH in a mixture of MeOH/H_2_O at rt overnight. The desired **PFK15** inhibitor (**6)** was finally synthetized from commercially available 2-quinolinecarboxaldehyde (**5**) and the previously prepared compound **4** in refluxing toluene overnight, with 78% yield (or 36.5% overall yield via 4 steps). **PFK15** was purified by crystallization from EtOH or ethyl acetate (EA).

#### 4.1.3. Synthesis of 2-Bromo-1-(pyridin-4-yl)ethan-1-one hydrobromide (**2**)

A solution of 32.0 g (264 mmol, 1.00 mol eq) 1-(pyridin-4-yl)ethan-1-one (**1**) in 250 mL glacial acetic acid (100%) was cooled in an ice bath, and 150 mL conc. HBr (48 *w*/*w* %) was carefully added. Subsequently, a solution of 42.2 g (264 mmol, 1.00 mol eq) Br_2_ in 20 mL glacial acetic acid was added dropwise to the reaction mixture. The reaction was stirred overnight at rt while a white precipitate formed. Afterwards, 250 mL Et_2_O was added, and the mixture was stirred for 30 min at rt and filtered. The obtained solid material was washed with 150 mL Et_2_O, dried to yield 70.0 g (249 mmol, 94%) of **2** in the form of a white solid powder. Salt **2** was used in the next step without further purification. Its m.p., ^1^H-NMR [24] and MS [25] spectra are described in the literature.

#### 4.1.4. Synthesis of (2-Oxo-2-(pyridin-4-yl)ethyl)triphenylphosphonium bromide (**3**)

First of all, 65.4 g (249 mmol, 1.00 mol eq) PPh_3_ and 34.7 mL (25.2 g, 249 mmol, 1.00 mol eq) Et_3_N were sequentially added to a solution of 70.0 g (249 mmol, 1.00 mol eq) **2** in 400 mL of THF. The reaction mixture was refluxed overnight while an orange precipitate formed. After cooling the mixture to rt, the solid material was filtered off, washed with 150 mL Et_2_O, and dried under reduced pressure to yield 95.9 g (207 mmol, 83%) of an orange product **3**, which was used without further purification in the next reaction.

#### 4.1.5. Synthesis of 1-(Pyridin-4-yl)-2-(triphenyl-λ^5^-phosphanylidene)ethan-1-one (**4**)

Within 30 min, 10.6 g (265 mmol, 3.00 mol eq) NaOH in 303 mL H_2_O was added to a solution of 40.9 g (88.5 mmol, 1.00 mol eq) **3** in a mixture of solvents (82 mL of MeOH and 151 mL of H_2_O). The mixture was stirred overnight at rt. The brown precipitate was filtered off, and washed sequentially with 100 mL H_2_O and 100 mL Et_2_O and dried under reduced pressure. A yield of 20.11 g (52.7 mmol, 60%) of **4** was obtained and used without further purification in the next step. Its m.p., ^1^H-NMR, and IR spectra are described in the literature [26].

#### 4.1.6. Synthesis of (*E*)-1-(Pyridin-4-yl)-3-(quinolin-2-yl)prop-2-en-1-one (**6**, **PFK15**)

First of all, 8.86 g (23.2 mmol, 1.00 mol eq) of a previously prepared compound **4** was added to a solution of 3.65 g (23.2 mmol, 1.00 mol eq) 2-quinolinecarboxaldehyde (**5**) in 250 mL toluene. The reaction mixture was stirred and refluxed overnight while a precipitated material appeared in the reaction. The mixture was cooled down and volatile parts were evaporated by rotary vacuum evaporator (RVE). The obtained crude reaction product (4.71 g, 78%) was crystallized from EtOH, yielding 2.54 g (9.76 mmol, 42%) of the required inhibitor **6** (**PFK15**), possessing an HPLC-UV (254 nm) purity of 99.2%. The synthesis and other characteristics of compound **6** are not yet described in the literature.

**M.p.:** 159.4–160.6 °C [EtOH].

**Figure 4:**
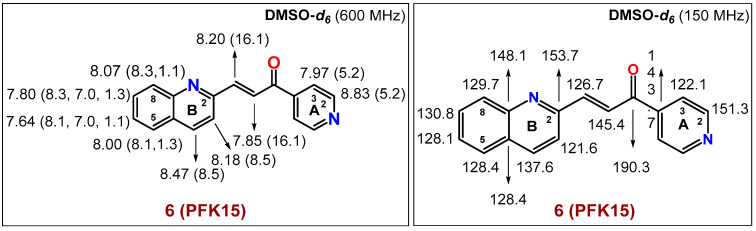
The spectral diagrams describing ^1^H- and ^13^C-NMR characteristics of **6** (**PFK15**).

The spectral diagrams (^1^H- and ^13^C-NMR) of **6** (**PFK15**) are based on 1D- and 2D-NMR techniques and are shown in Figure 4 below (see also the Supplementary Material to this paper).

**^1^H-NMR** (600 MHz, DMSO-*d*_6_): *δ* 8.83 (d, 2H, *J*(A_2_,A_3_) = 5.2 Hz, 2 × H-C_A_(2)), 8.47 (d, 1H, *J*(B_3_,B_4_) = 8.5 Hz, H-C_B_(4)), 8.20 (d, 1H, *J*(CH=CH) = 16.1 Hz, -CH=CH-C=O), 8.18 (d, 1H, *J*(B_3_,B_4_) = 8.5 Hz, H-C_B_(3)), 8.07 (dd, 1H, *J*(B_7_,B_8_) = 8.3 Hz, *J*(B_6_,B_8_) = 1.1 Hz, H-C_B_(8)), 8.00 (dd, 1H, *J*(B_5_,B_6_) = 8.1 Hz, *J*(B_5_,B_7_) = 1.3 Hz, H-C_B_(5)), 7.97 (d, 2H, *J*(A_2_,A_3_) = 5.2 Hz, 2 × H-C_A_(3)), 7.85 (d, 1H, *J*(CH=CH) = 16.1 Hz, -CH=CH-C=O), 7.80 (ddd, 1H, *J*(B_7_,B_8_) = 8.3 Hz, *J*(B_6_,B_7_) = 7.00 Hz, *J*(B_5_,B_7_) = 1.3 Hz, H-C_B_(7)), 7.64 (ddd, 1H, *J*(B_5_,B_6_) = 8.1 Hz, *J*(B_6_,B_7_) = 7.00 Hz, *J*(B_6_,B_8_) = 1.1 Hz, H-C_B_(6)).

**^13^C-NMR** (150 MHz, DMSO-*d*_6_): *δ* 190.3 (-C=O), 153.7 C_B_(2), 151.3 (2 × C_A_(2)), 148.1 C_B_(9), 145.4 (-CH=CH-C=O), 143.7 C_A_(4), 137.6 C_B_(4), 130.8 C_B_(7), 129.7 C_B_(8), 128.4 C_B_(10), 128.4 C_B_(5), 128.1 C_B_(6), 126.7 (-CH=CH-C=O), 122.1 (2 × C_A_(3)), 121.6 C_B_(3).

**FT IR** (solid sample, cm^−1^): 3051 (m), 2111 (w), 2054 (w), 1930 (w), 1858 (w), 1661 (m), 1589 (s), 1551 (m), 1502 (m), 1433 (w), 1408 (m), 1377 (w), 1351 (m), 1306 (m), 1286 (m), 1255 (m), 1209 (s), 1149 (m), 1117 (m), 1060 (w), 1034 (s), 980 (s), 951 (m), 897 (m), 872 (m), 842 (m), 820 (s), 877 (s), 770 (s), 738 (s), 698 (s), 655 (s), 554 (m), 524 (m), 495 (m), 477 (m), 448 (m), 428 (m).

**MS** of **6** (**PFK15**, calculated FW_exact_ = 260.09; ESI/positive mode: found [M+H]^+^ = 261.1).

**Elemental Analysis:** Calculated for C_17_H_12_N_2_O (260.30): C, 78.44; H, 4.65; N, 10.76. Found: C, 78.12; H, 4.38; N, 10.99.

The spectra and detailed spectral characteristics (1D-, 2D-NMR: NOESY, HSQC, HMBC, IR and MS) for **PFK15** (**6**), not yet published in the literature, are presented in the Supplementary Material to this paper (Figures S1–S7).

### 4.2. Biology

#### 4.2.1. Cell Culture and Cultivation

Human umbilical vein endothelial cells (HUVEC) were isolated from fresh umbilical cords, as described previously [4], and cultured in endothelial cell growth medium (ECGM; PromoCell, Heidelberg, Germany) supplemented with 100 U/mL penicillin and 100 µg/mL streptomycin (Biosera, Nuaillé, France). Cells were incubated in a humidified incubator (Heal Force Bio-meditech, Qingdao, China) at standard conditions (in 5% CO_2_ at 37 °C). Cells were used between passages 1 and 6.

#### 4.2.2. Drug Preparation

Stock solutions of the multikinase inhibitor **sunitinib *L*-malate** (Pfizer, New York, NY, USA) (abbreviated to **sunitinib** or **SU** in the text for simplification) and the glucose metabolism inhibitor (*E*)-1-(pyridin-4-yl)-3-(quinolin-2-yl)prop-2-en-1-one (**PFK15**) were prepared in dimethyl sulfoxide (DMSO; Sigma-Aldrich, USA) at a concentration of 10 mM. The stock solutions were subsequently diluted in an appropriate concentration in ECGM (and finally in test solutions containing 1% DMSO) for the in vitro experiments. **Sunitinib** was obtained as a gift from a pharmaceutical company (Pfizer Inc., New York, NY, USA). The synthesis and exact structure assignments of **PFK15** were performed as described above.

#### 4.2.3. IC_50_ Evaluation

Cells were seeded in a 96-well plate with serial dilutions of **PFK15** and **sunitinib** (starting at 100 µM solution containing 1% DMSO) at a density of 3 × 10^3^ cells/well. The MTS assay was performed according to the manufacturer’s instructions, as previously described [8]. The half maximal inhibitory concentration (IC_50_) value was calculated using Graph Pad Prism 6 software (Graph Pad, San Diego, CA, USA).

#### 4.2.4. Cell Proliferation Assay

The MTS assay is generally used for the quantification of cell proliferation, viability, or cytotoxicity. Cell proliferation was determined by colorimetric MTS assay, according to the manufacturer´s instructions. Briefly, cells were seeded in a 96-well plate at a density of 3 × 10^3^ cells/well and incubated with serial dilutions of **PFK15** and **sunitinib** for 3 days. After 3 days, 10 µL of yellow MTS (5 mg/mL) (Promega, Madison, WI, USA, CAS: 138169-43-4) was added to each well and cells were incubated for 2–3 h at 37 °C. The absorbances were measured at a wavelength of 490 nm (green color). All determinations were performed in quadruplicate, with three independent experiments.

#### 4.2.5. Migration Assay (Wound Healing Assay)

Cells were seeded in 24-well plates coated with 1.5% gelatin (Sigma-Aldrich, USA) at a density of 5 × 10^5^ cells/well (HUVEC). After reaching a confluent monolayer the medium was replaced with a starvation medium (ECGM supplemented with 2% FBS) and cells were incubated for a further 17 h. The confluent cell monolayer was wounded using pipet tips and washed twice with PBS. Subsequently, cells were treated with different doses of inhibitors diluted in ECGM medium. The migration of HUVEC was observed with an Olympus IMT2 inverted optical microscope (Olympus, Tokyo, Japan) and recorded by a Moticam 1000 camera system (Motic Incorporation, Hong Kong) at 0 h and 8 h after treatment. Changes in cell migration were evaluated using the software Motic Images Plus 2.0 PL (Motic Incorporation, Hong Kong).

### 4.3. Statistical Analysis

The results are expressed as a mean ± SEM. Each value represents the average of at least three independent experiments. Statistical analysis was performed using STATISTICA 7.0 (StatSoft Inc., Tulsa, OK, USA) and GraphPad Prism 6 (GraphPad Software, Inc.). Statistical differences among groups were determined by one-way ANOVA followed by Tukey’s post hoc test. The value *p* < 0.05 was considered as significant.

## 5. Conclusions

In conclusion, we described the synthesis of the glycolysis inhibitor PFK15 in detail. Our results confirmed the effectivity of PFK15 in combination with sunitinib. **Sunitinib** is an approved drug with therapeutic applications against tumor growth and tumor angiogenesis. The glycolysis inhibitor **PFK15** reduces glucose uptake and causes starvation of activated endothelial and tumor cells. Both tumor and activated endothelial cells are dependent on a high influx of glucose as an important source of energy and building blocks. Therefore, the synergistic effect of the above inhibitors to block different biological targets and mechanisms of action on HUVEC-based angiogenesis is promising from a therapeutical point of view.

## Figures and Tables

**Table 1:** The IC_50_ values for **PFK15** and **sunitinib** determined on HUVEC.

	IC_50_ [µM]
**PFK15**	2.6
**sunitinib**	1.6

## Supplementary Materials

The following supporting information can be downloaded at: https://www.mdpi.com/article/10.3390/ijms232214295/s1.
