# Supplementary material for: Synthesis of Glycolysis Inhibitor PFK15 and Its Synergistic Action with an Approved Multikinase Antiangiogenic Drug on Human Endothelial Cell Migration and Proliferation
Source: Int J Mol Sci. 2022 Nov 18;23(22):14295. doi: 10.3390/ijms232214295 (PMC9697023; doi:10.3390/ijms232214295)
Supplement: Supplementary file 1 [file ijms-23-14295-s001.zip › ijms-1969006-supplementary.pdf]

Supplementary Material

Synthesis of Glycolysis Inhibitor PFK15 and Its Synergistic Action with an Approved Multikinase Antiangiogenic Drug on Human Endothelial Cell Migration and Proliferation

Jana Zlacká [1], Miroslav Murár [2], Gabriela Addová [2], Roman Moravčík [1], Andrej Boháč [2,3,†] and Michal Zeman [1,*]

[1] Department of Animal Physiology and Ethology, Faculty of Natural Sciences, Comenius University in Bratislava, Ilkovičova 6, 842 15 Bratislava, Slovakia
[2] Department of Organic Chemistry, Faculty of Natural Sciences, Comenius University in Bratislava, Ilkovičova 6, 842 15 Bratislava, Slovakia
[3] Biomagi, Ltd., Mamateyova 26, 851 04 Bratislava, Slovakia
* Correspondence: michal.zeman@uniba.sk
† Author responsible for the chemistry part of this work (synthesis and description of PFK15).

The synthesis of **PFK15**

The spectra and detailed spectral characteristics (1D-, 2D-NMR: NOESY, HSQC, HMBC, IR and MS) for **PFK15 (6)** are on **Figures S1-S7** and below.

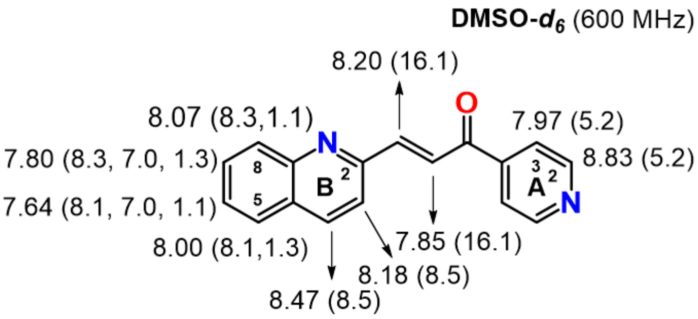

6 (PFK15)

^{1}**H-NMR** (600 MHz, DMSO-d_6): δ 8.83 (d, 2H, *J*(A$_2$,A$_3$) = 5.2 Hz, 2 x H-C$_A$(2)), 8.47 (d, 1H, *J*(B$_3$,B$_4$) = 8.5 Hz, H-C$_B$(4)), 8.20 (d, 1H, *J*(CH=CH) = 16.1 Hz, -C<u>H</u>=CH-C=O), 8.18 (d, 1H, *J*(B$_3$,B$_4$) = 8.5 Hz, H-C$_B$(3)), 8.07 (dd, 1H, *J*(B$_7$,B$_8$) = 8.3 Hz, *J*(B$_6$,B$_8$) = 1.1 Hz, H-C$_B$(8)), 8.00 (dd, 1H, *J*(B$_5$,B$_6$) = 8.1 Hz, *J*(B$_5$,B$_7$) = 1.3 Hz, H-C$_B$(5)), 7.97 (d, 2H, *J*(A$_2$,A$_3$) = 5.2 Hz, 2 x H-C$_A$(3)), 7.85 (d, 1H, *J*(CH=CH) = 16.1 Hz, -CH=C<u>H</u>-C=O), 7.80 (ddd, 1H, *J*(B$_7$,B$_8$) = 8.3 Hz, *J*(B$_6$,B$_7$) = 7.00 Hz, *J*(B$_5$,B$_7$) = 1.3 Hz, H-C$_B$(7)), 7.64 (ddd, 1H, *J*(B$_5$,B$_6$) = 8.1 Hz, *J*(B$_6$,B$_7$) = 7.00 Hz, *J*(B$_6$,B$_8$) = 1.1 Hz, H-C$_B$(6)).

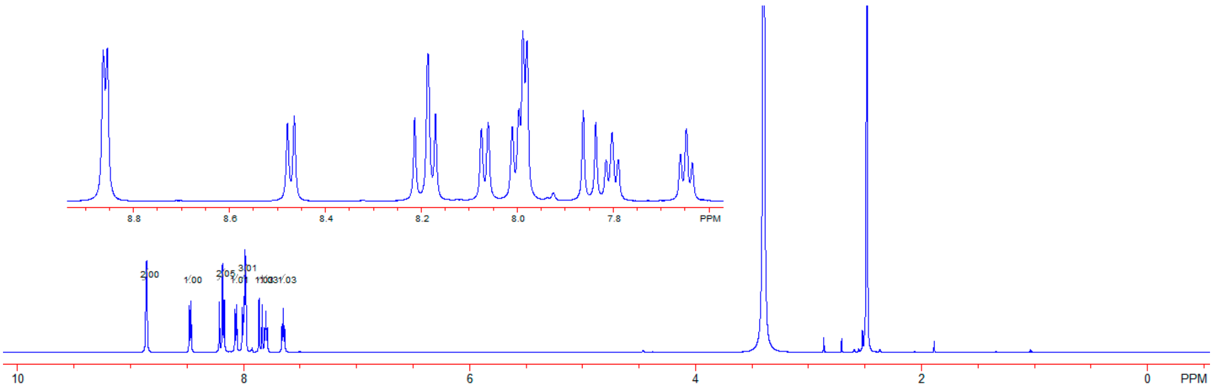

Figure S1: ^{1}H-NMR (600 MHz, DMSO-d_6) spectrum of compound **6 (PFK15)**.

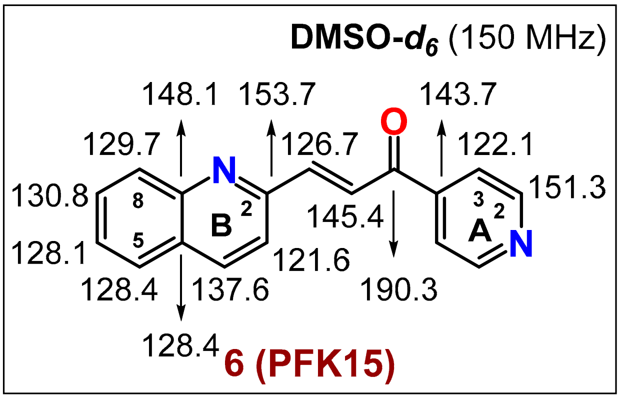

^{13}C-NMR (150 MHz, DMSO-d_6): δ 190.3 (-C=O), 153.7 C_B(2), 151.3 (2 x C_A(2)), 148.1 C_B(9), 145.4 (-CH=CH-C=O), 143.7 C_A(4), 137.6 C_B(4), 130.8 C_B(7), 129.7 C_B(8), 128.4 C_B(10), 128.4 C_B(5), 128.1 C_B(6), 126.7 (-CH=CH-C=O), 122.1 (2 x C_A(3)), 121.6 C_B(3).

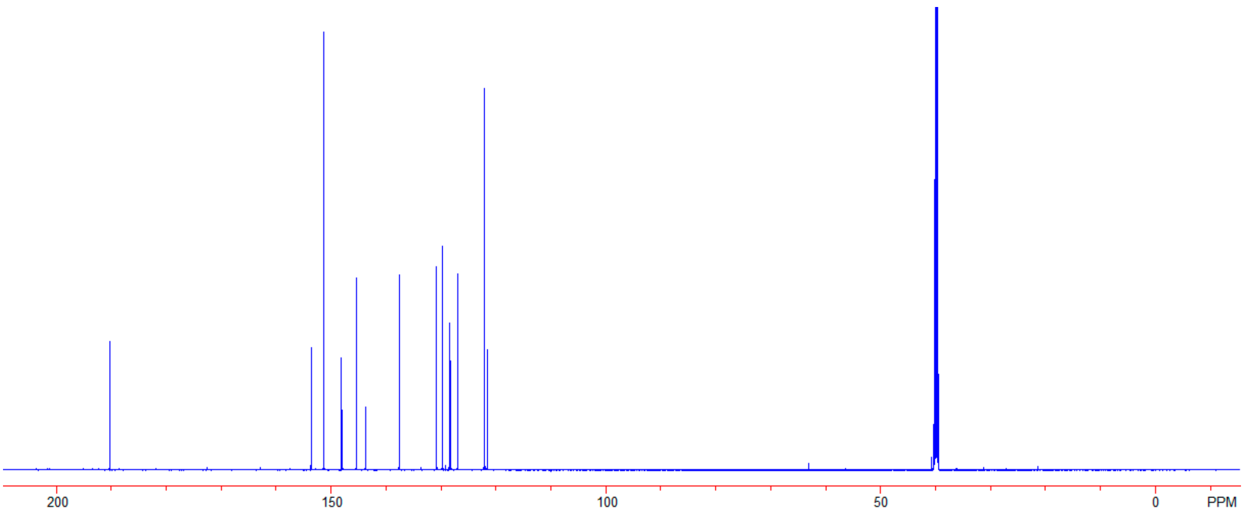

Figure S2: ^{13}C-NMR (150 MHz, DMSO-d_6) spectrum of compound **6 (PFK15)**.

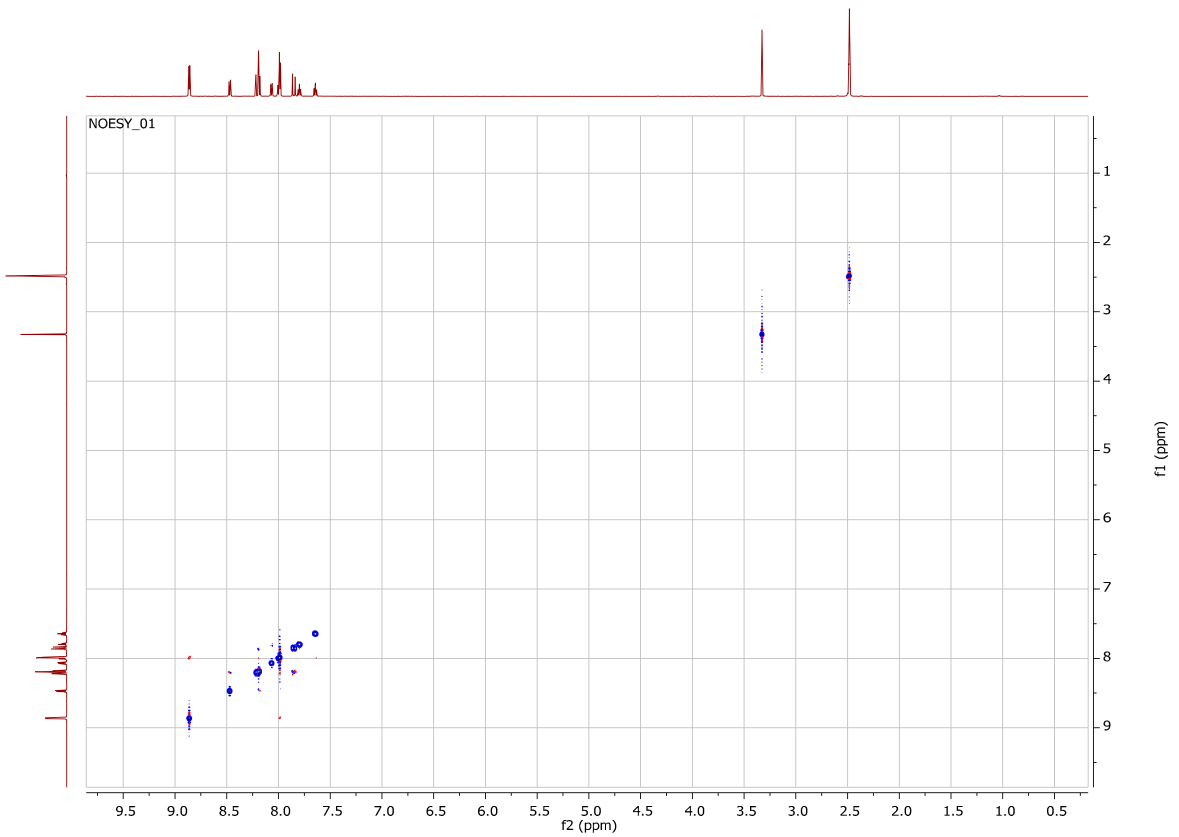

Figure S3: NOESY spectrum of **6 (PFK15)**.

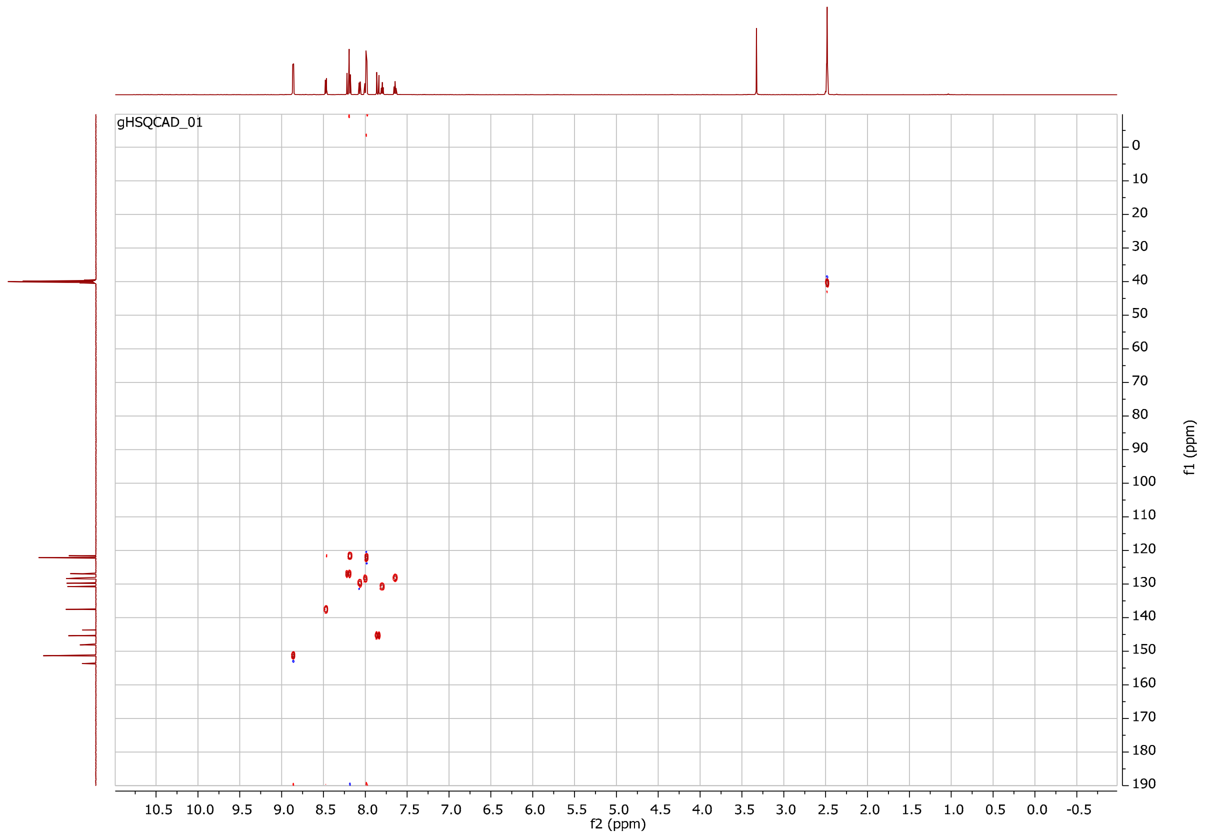

Figure S4: HSQC spectrum of **6 (PFK15).**

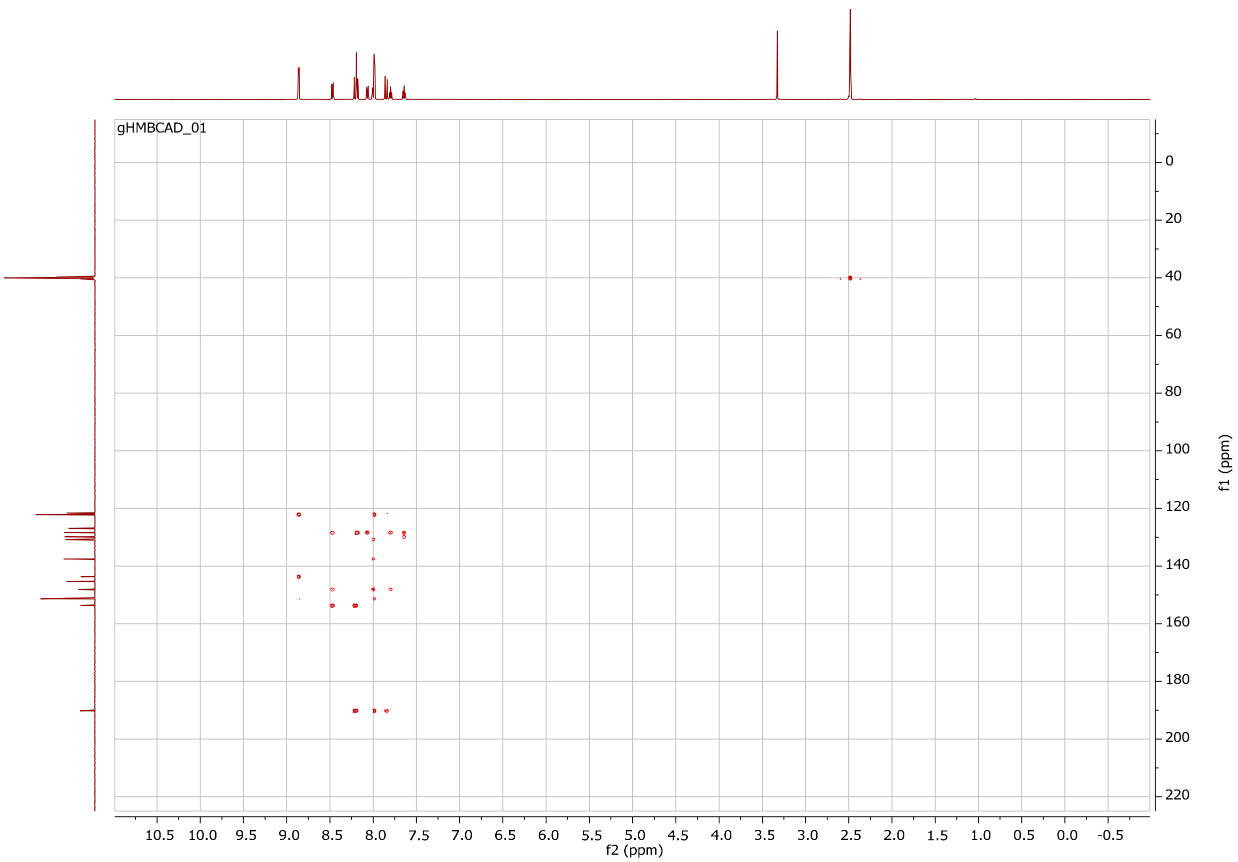

Figure S5: HMBC spectrum of **6 (PFK15)**.

FT IR (solid sample, cm^{-1}): 3051 (m), 2111 (w), 2054 (w), 1930 (w), 1858 (w), 1661 (m), 1589 (s), 1551 (m), 1502 (m), 1433 (w), 1408 (m), 1377 (w), 1351 (m), 1306 (m), 1286 (m), 1255 (m), 1209 (s), 1149 (m), 1117 (m), 1060 (w), 1034 (s), 980 (s), 951 (m), 897 (m), 872 (m), 842 (m), 820 (s), 877 (s), 770 (s), 738 (s), 698 (s), 655 (s), 554 (m), 524 (m), 495 (m), 477 (m), 448 (m), 428 (m).

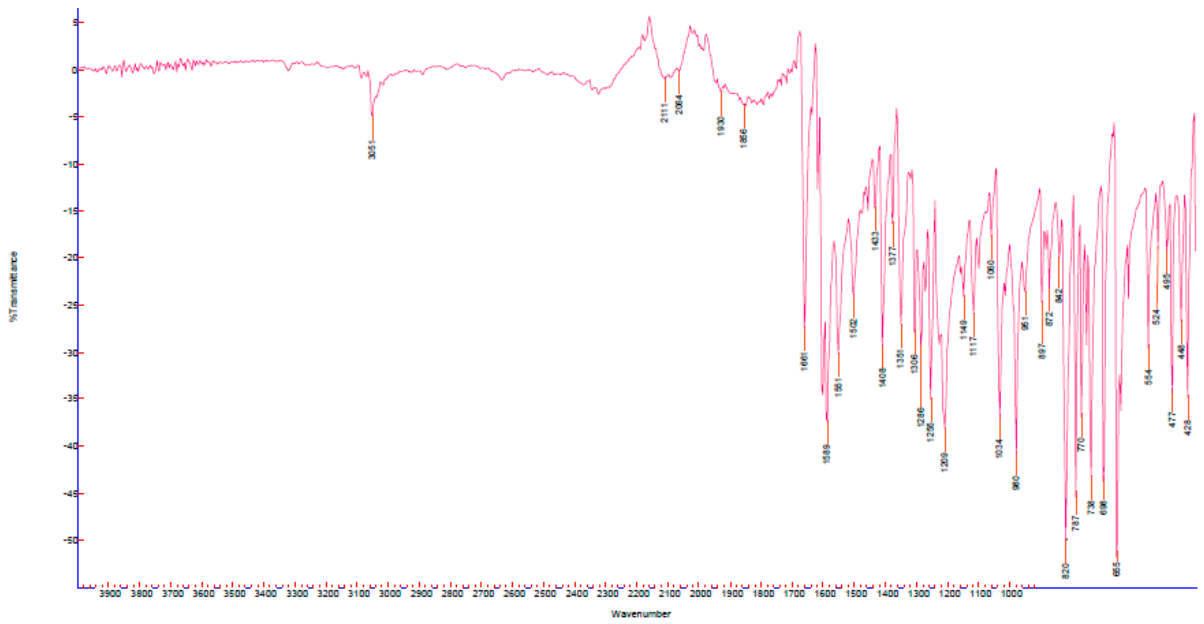

Figure S6: IR (solid sample, cm^{-1}) spectrum of **6 (PFK15)**.

MS (ESI m/z): 261.1 $[M+H]^+$.

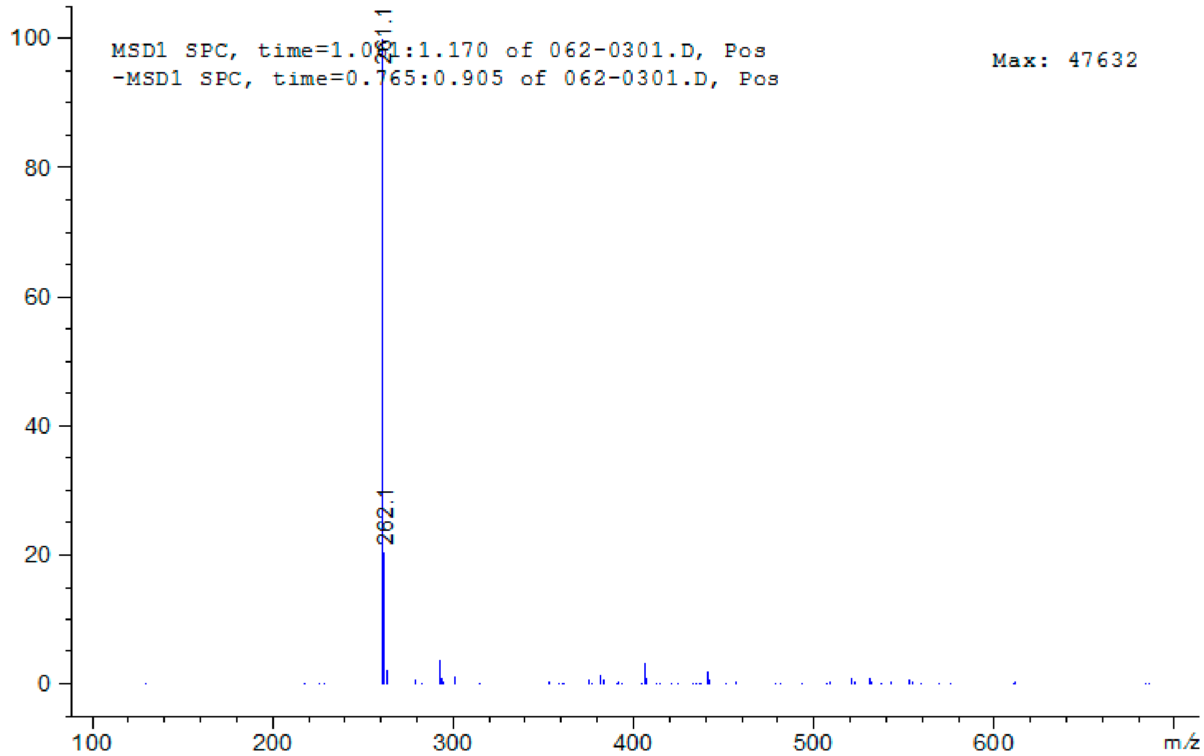

Figure S7: MS (ESI, positive mode) spectrum of **6** (**PFK15**, FW_{exact} = 260.09).

Elemental Analysis: Calculated for $C_{17}H_{12}N_2O$ (260.30): C, 78.44; H, 4.65; N, 10.76. Found: C, 78.12; H, 4.38; N, 10.99.

Effect of **PFK15** and **sunitinib** on HUVEC migration (wound healing assay)

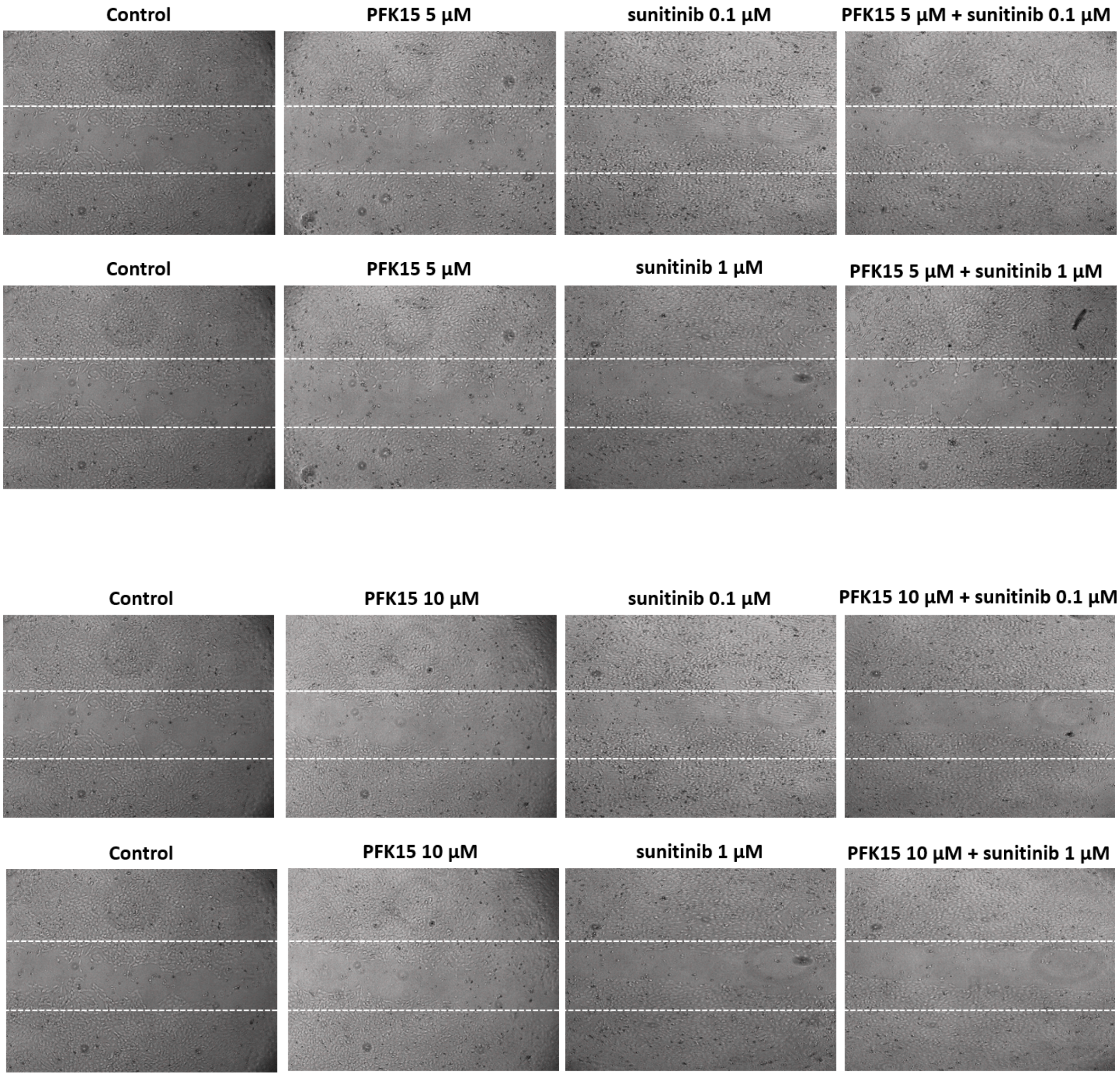

Figure S8: Evaluation of administration of **PFK15** ((*E*)-1-(pyridin-4-yl)-3-(quinolin-2-yl)prop-2-en-1-one) and **sunitinib** alone or in combination on HUVEC migration. Cells were observed with an inverted optical microscope Olympus IMT 2 and evaluated with camera system Moticam 1000 at 40 x magnification. The areas marked by white lines show the initially damaged monolayer of cells at time 0 and the same area 8 hours after the treatment.

Effect of **PFK15** and **sunitinib** on HUVEC proliferation

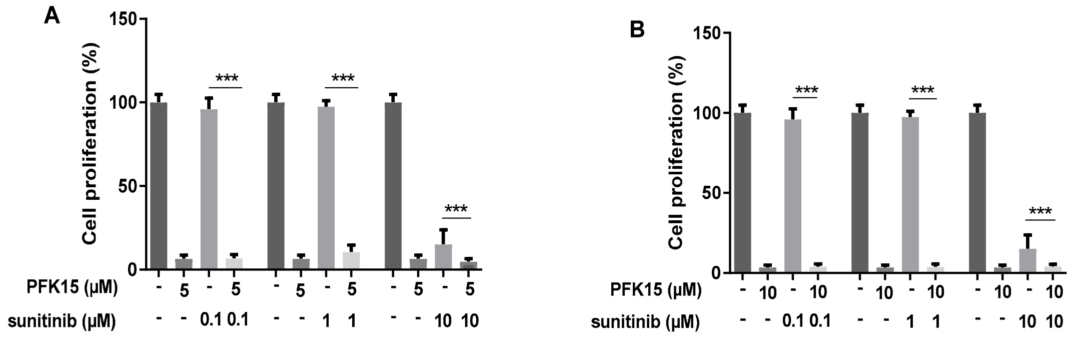

Figure S9: Evaluation of administration of **PFK15** ((*E*)-1-(pyridin-4-yl)-3-(quinolin-2-yl)prop-2-en-1-one) and **sunitinib** alone or in combination on HUVEC proliferation. HUVECs were treated with **PFK15** at 5 or 10 µM and **sunitinib** at 0.1, 1.0 or 10 µM for 72 hours and changes in cell proliferation were measured. Data are presented as mean ± SEM of three independent experiments (***$p < 0.001$).
